# Connectome analysis of functional and structural hemispheric brain networks in major depressive disorder

**DOI:** 10.1038/s41398-019-0467-9

**Published:** 2019-04-12

**Authors:** Xueyan Jiang, Yuedi Shen, Jiashu Yao, Lei Zhang, Luoyi Xu, Rui Feng, Liqiang Cai, Jing Liu, Wei Chen, Jinhui Wang

**Affiliations:** 1 Institute for Brain Research and Rehabilitation, Guangdong Key Laboratory of Mental Health and Cognitive Science, Center for Studies of Psychological Application, South China Normal University, Guangzhou, China; 2 Department of Diagnostics, Clinical Medical School, Hangzhou Normal University, 310036 Hangzhou, Zhejiang China; 3 Department of Psychiatry, Sir Run Run Shaw Hospital, Zhejiang University School of Medicine, Key Laboratory of Medical Neurobiology of Zhejiang Province, 310016 Hangzhou, Zhejiang China

## Abstract

Neuroimaging studies have shown topological disruptions of both functional and structural whole-brain networks in major depressive disorder (MDD). This study examined common and specific alterations between these two types of networks and whether the alterations were differentially involved in the two hemispheres. Multimodal MRI data were collected from 35 MDD patients and 35 healthy controls, whose functional and structural hemispheric networks were constructed, characterized, and compared. We found that functional brain networks were profoundly altered at multiple levels, while structural brain networks were largely intact in patients with MDD. Specifically, the functional alterations included decreases in intra-hemispheric (left and right) and inter-hemispheric (heterotopic) functional connectivity; decreases in local, global and normalized global efficiency for both hemispheric networks; increases in normalized local efficiency for the left hemispheric networks; and decreases in intra-hemispheric integration and inter-hemispheric communication in the dorsolateral superior frontal gyrus, anterior cingulate gyrus and hippocampus. Regarding hemispheric asymmetry, largely similar patterns were observed between the functional and structural networks: the right hemisphere was over-connected and more efficient than the left hemisphere globally; the occipital and partial regions exhibited leftward asymmetry, and the frontal and temporal sites showed rightward lateralization with regard to regional connectivity profiles locally. Finally, the functional–structural coupling of intra-hemispheric connections was significantly decreased and correlated with the disease severity in the patients. Overall, this study demonstrates modality- and hemisphere-dependent and invariant network alterations in MDD, which are helpful for understanding elaborate and characteristic patterns of integrative dysfunction in this disease.

## Introduction

Major depressive disorder (MDD) is a chronic, recurrent illness characterized by significant suffering, high morbidity, and mortality rates and psychosocial impairments^1^. Accumulating evidences from neuroimaging studies have shown that MDD is associated with abnormal topological organization of functional and structural brain networks, such as altered network efficiency^2,3^ and regional centrality^4,5^. Thus, MDD is increasingly recognized as a network dysfunctional disease^6^.

Human brain networks can be derived by estimating interregional neural synchronization functionally with functional MRI^7^ and reconstructing fiber pathway structurally with diffusion MRI^8^. It has been well documented that both functional and structural brain networks share many organizational principles in favor of efficient signaling, information exchange and processing, such as small-worldness, modularity, and highly connected hubs^9–13^. Although it is universally acknowledged that functional connectivity patterns are largely constrained by the underlying structural connectivity layouts^8,14–16^, recent studies indicate that there lacks a one-to-one correspondence between these two types of networks^16,17^. Accordingly, simultaneous analyses of functional and structural networks could provide complementary insights into brain organization under both healthy and pathological conditions^18^. Regarding MDD, however, few studies have examined common and specific topological alterations between functional and structural networks. Moreover, the combination of functional and structural networks provides another benefit that allows for an examination of functional–structural coupling, which is a clinically meaningful index for exploring brain diseases^18–20^.

Another factor that may affect the brain network organization is the hemisphere. Recently, several studies consistently show that the topological organizations of functional and structural networks are not uniform across brain hemispheres^21–23^. The hemispheric asymmetry of network organization may underlie the functional specialization of different cognitive functions of the human brain. Moreover, altered brain network asymmetry is linked to development processes^24^ and neuropsychiatric disorders^25^. For MDD, although abnormal brain asymmetries in local brain features of specific regions have been documented in previous studies^6^, it remains largely unknown whether the two hemispheres are distinctively involved in the disease at the network level.

In this study, we investigated MDD-related brain network alterations by taking network modality and brain hemisphere into account. Specifically, we utilized resting-state functional MRI (R-fMRI) and diffusion tensor imaging (DTI) to construct functional and structural hemispheric networks for 35 patients with MDD and 35 healthy controls (HCs). Graph-based network approaches were then used to topologically characterize these hemispheric networks at the levels of overall wiring patterns, global network organization and local regional roles. Finally, functional–structural coupling and brain-clinical relationships were examined. We hypothesized that MDD is related to disrupted network architecture in a modality- and hemisphere-dependent manner.

## Materials and methods

### Participants

All participants included in the current study, including 35 patients with MDD and 35 HCs, were screened from an ongoing follow-up project that aims to explore the relationships between baseline brain architecture and clinical outcomes of MDD patients after antidepressant treatment using multimodal MRI data. MDD was diagnosed according to the Diagnostic and Statistical Manual of Mental Disorders, Fourth Edition, Text Revision (DSM-IV-TR) criteria, using the Structured Clinical Interview for the DSM-IV (SCID)-I. The exclusion criteria included (1) severe suicidal tendency; (2) pregnant or lactating women; (3) physical diseases as assessed by personal history; (4) a history of organic brain disorders, neurological disorders, other psychiatric disorders, or cardiovascular diseases; and (5) a history of substance abuse, including tobacco, alcohol, or other psychoactive substances. All patients had a 17-item Hamilton Depression Scale (HAMD) score ≥ 18 and a Mood Disorder Questionnaire (MDQ) score < 7 and were free of psychotropic medications for at least 4 weeks before the baseline MRI scan. The MDD patients were recruited from outpatients and inpatients of the Sir Run Run Shaw Hospital, School of Medicine, Zhejiang University, Hangzhou, China, and the HCs were recruited from the local community via advertisement. The detailed demographic and clinical characteristics for all participants are summarized in Supplementary Table 1. Datasets from a subset of the population were used in our previous study^19,20^. This study was approved by the Ethics Committee of the Sir Run Run Shaw Hospital, School of Medicine, Zhejiang University, and the Affiliated Hospital of Hangzhou Normal University. All participants gave written informed consent.

### MRI data acquisition

All MRI data were acquired on a 3.0T MR scanner (GE Discovery MR750, GE Medical Systems, Milwaukee, WI) with an eight-channel head coil array. During the scanning, all participants were instructed to lie quietly in the scanner with their eyes open and to try not to think of anything systematically. See Supplementary Material for the detailed imaging parameters.

### Data preprocessing and network construction

Before constructing functional and structural brain networks, the R-fMRI and DTI data were first preprocessed using GRETNA^26^ based on SPM12 (http://www.fil.ion.ucl.ac.uk/spm/software/spm12/) and PANDA (https://www.nitrc.org/projects/panda/) based on FSL (http://fsl.fmrib.ox.ac.uk/fsl/fslwiki/), respectively. Details of the processing flows are described in the Supplementary Material. Then, we constructed individual functional and structural brain networks at the macroscale, which were composed of nodes and edges with nodes representing brain regions and edges representing interregional functional or structural connectivity. To define network nodes, we employed an automated anatomical labeling atlas^27^ to parcel the cerebrum into 90 regions of interest (45 in each hemisphere) (Supplementary Table 2). To define network edges, we calculated pairwise Pearson correlation coefficients for the R-fMRI data and pairwise fiber numbers for the DTI data among the 90 regions. These procedures resulted in two whole-brain 90 × 90 weighted matrices for each participant. After applying a connectivity strength-based thresholding procedure to each matrix, two 45 × 45 hemispheric networks were finally obtained by eliminating all inter-hemispheric connections.

### Graph-based network analysis

Prior to topological analysis of the hemispheric networks derived above, all connections were categorized into intra-hemispheric connections (left and right) and inter-hemispheric connections (homotopic: edges linking geometrically corresponding regions between the two hemispheres; heterotopic: edges linking geometrically non-corresponding regions between the two hemispheres), and the mean connectivity strength of each category was calculated. Then, we calculated four global (global efficiency, local efficiency, normalized global efficiency, and normalized local efficiency) and two regional (intra-hemispheric nodal degree and inter-hemispheric nodal degree) network measures for each hemispheric network. See Supplementary Material for the formulas and an excellent review^28^ for the uses and interpretations of these network measures.

### Functional–structural coupling of hemispheric brain networks

To examine the functional–structural coupling of intra- and inter-hemispheric connectivity patterns, we first counted the number (i.e., coupling amount) of the region pairs that were functionally and structurally connected simultaneously. Then, for these connections, we further calculated their (cross-connection) Pearson correlation (i.e., coupling degree) between functional weights (i.e., interregional correlation coefficients) and structural weights (i.e., interregional fiber numbers). For the correlation analysis, the functional weights were transformed into z values using Fisher’s r-to-z transformation, and the structural weights were resampled into a normal distribution^16,29^.

### Statistical analysis

#### Between-group difference

For the between-group differences in demographic variables, independent two-sample *t*-tests were used for age and education, and a chi-squared test was used for gender. For MRI-based network metrics, inter-hemispheric connectivity (homotopic and heterotopic), and the functional–structural coupling of inter-hemispheric connectivity were tested with independent two-sample *t*-tests. Other network measures were examined using two-way mixed ANOVA, including the intra-hemispheric connectivity, global efficiency, local efficiency, normalized global efficiency, normalized local efficiency, nodal degree (intra- and inter-hemispheric), and functional–structural coupling of intra-hemispheric connectivity. For the two-way mixed ANOVA, group was a between-subject factor, and hemisphere was a within-subject factor. If any effect survived a threshold of *P* < 0.05 (Bonferroni-corrected for nodal degree), post hoc tests were further performed (paired *t*-test for the hemisphere effect and independent *t*-test for the group effect). For all statistical comparisons of MRI-based network measures, age, gender, and education were treated as covariates (with the exception of post hoc comparisons between hemispheres). Mean framewise displacement of head motion was added as an additional covariate for functional comparisons. All statistical analyses were performed using the MATLAB or R software.

#### Relationship between network measures and clinical variables

Partial correlation analysis was used to examine the relationship between MDD-related network alterations and clinical variables (HAMD score and disease duration) of the patients after controlling for confounding effects of age, gender, and education. Multiple comparisons were not corrected due to the relatively small sample size and exploratory nature of the correlation analysis.

### Validation analysis

We performed the following validation analyses to test the reproducibility of our main results.

#### Regional size

Previous studies have shown that regional size may bias the estimation of nodal connectivity for both functional and structural brain networks^8,30^. To examine the possible effects of this factor on our results, we performed two-way mixed ANOVA to test whether the Pearson correlations (Fisher r-to-z transformed) between regional size (surface area for the structural networks and volume for the functional networks) and nodal degree (intra- and inter-hemispheric) of each network modality differed between the groups or were dependent on the hemispheres. If significant effects were observed, we further calculated the normalized nodal degree (by corresponding regional size) for between-group comparisons.

#### Thresholding procedure

In this study, we utilized a connectivity strength-based thresholding procedure to investigate MDD-related alterations in the absolute network organization of functional and structural networks. However, this type of thresholding procedure can lead to different network densities across participants, which may confound subsequent between-group comparison^31^. Thus, we also re-analyzed our data by forcing the same network density (0.08–0.15, interval = 0.01) and the same level of overall connectivity strength (overall mean = 1) across participants. By correcting for different network densities and overall connectivity strengths, this type of thresholding procedure enables examinations of MDD-related alterations in relative network organization^32^. Specifically, the minimum network density was selected to ensure that the mean degree was greater than 2 × log (*N*) for each resultant network, where *N* is the number of nodes of the hemispheric networks (i.e., 45). The maximum density was determined by the fewest number of edges among all thresholded hemispheric networks using the connectivity strength-based thresholding procedures. This approach avoids a modification of network topology by excluding spurious connections as much as possible. For the resultant networks, we calculated their global and nodal network attributes as described previously, whose areas under the curve (i.e., the integrals over different network densities) were used for subsequent statistical analyses.

## Results

### Demographics, clinical characteristics and head motion

No significant differences were found in age, gender, education, or head motion between the two groups (Supplementary Table 1) (*P* > 0.05).

### Intra- and inter-hemispheric connectivity

#### Functional networks

**Fig. 1:**
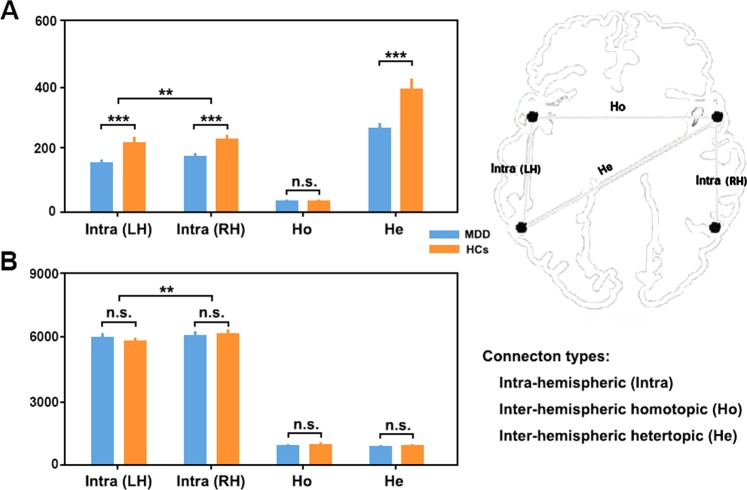
Intra- and inter-hemispheric connectivity. Alterations of intra- and inter-hemispheric connectivity for functional (**a**) and structural (**b**) brain networks. MDD major depressive disorder, HCs healthy controls, LH left hemisphere, RH right hemisphere, n.s. non-significant; **P < 0.01; ***P < 0.001

For the intra-hemispheric functional connectivity, significant group (*F*_1,68_ = 10.384, *P* = 0.002) and hemisphere (*F*_1,68_ = 12.067, *P* < 0.001) main effects were observed without group × hemisphere interaction (*F*_1,68_ = 1.078, *P* = 0.303). Post hoc analyses revealed that the main effects were driven by decreased connectivity in the MDD patients vs. HCs (MDD = 162.046 ± 50.908, HCs = 220.767 ± 84.235; *T*_133_ = 4.856, *P* < 0.001) and lower connectivity in the left vs. right hemisphere (left = 183.707 ± 74.750, right = 199.105 ± 75.687; *T*_69_ = 3.472, *P* < 0.001). For inter-hemispheric functional connectivity, significant decreases for heterotopic connections (MDD = 260.120 ± 96.606, HCs = 382.906 ± 171.144; *T*_63_ = 3.570, *P* < 0.001) and a trend toward significant decreases for homotopic connections (MDD = 30.760 ± 3.244, HCs = 32.359 ± 2.645; *T*_63_ = 1.935, *P* = 0.057) were found in the MDD patients compared with the HCs (Fig. 1a).

#### Structural networks

For the intra-hemispheric structural connectivity, only a significant hemisphere main effect was observed (*F*_1,68_ = 14.550, *P* < 0.001) due to lower connectivity in the left vs. right hemisphere (left = 5888.643 ± 953.629, right = 6125.686 ± 951.463; *T*_69_ = 3.768, *P* < 0.001). There was no significant group effect (MDD = 6016.929 ± 929.530, HCs = 5987.400 ± 989.128; *F*_1,68_ = 0.377, *P* = 0.541) or group × hemisphere interaction (*F*_1,68_ = 2.684, *P* = 0.106). For inter-hemispheric structural connectivity, no significant differences were found between the MDD patients and HCs for neither heterotopic (MDD = 860.771 ± 347.505, HCs = 875.600 ± 324.746; *T*_65_ = 0.612, *P* = 0.543) or homotopic (MDD = 871.771 ± 394.793, HCs = 918.600 ± 414.488; *T*_65_ = 0.379, *P* = 0.706) connections (Fig. 1b).

### Global hemispheric network efficiency

#### Functional networks

**Fig. 2:**
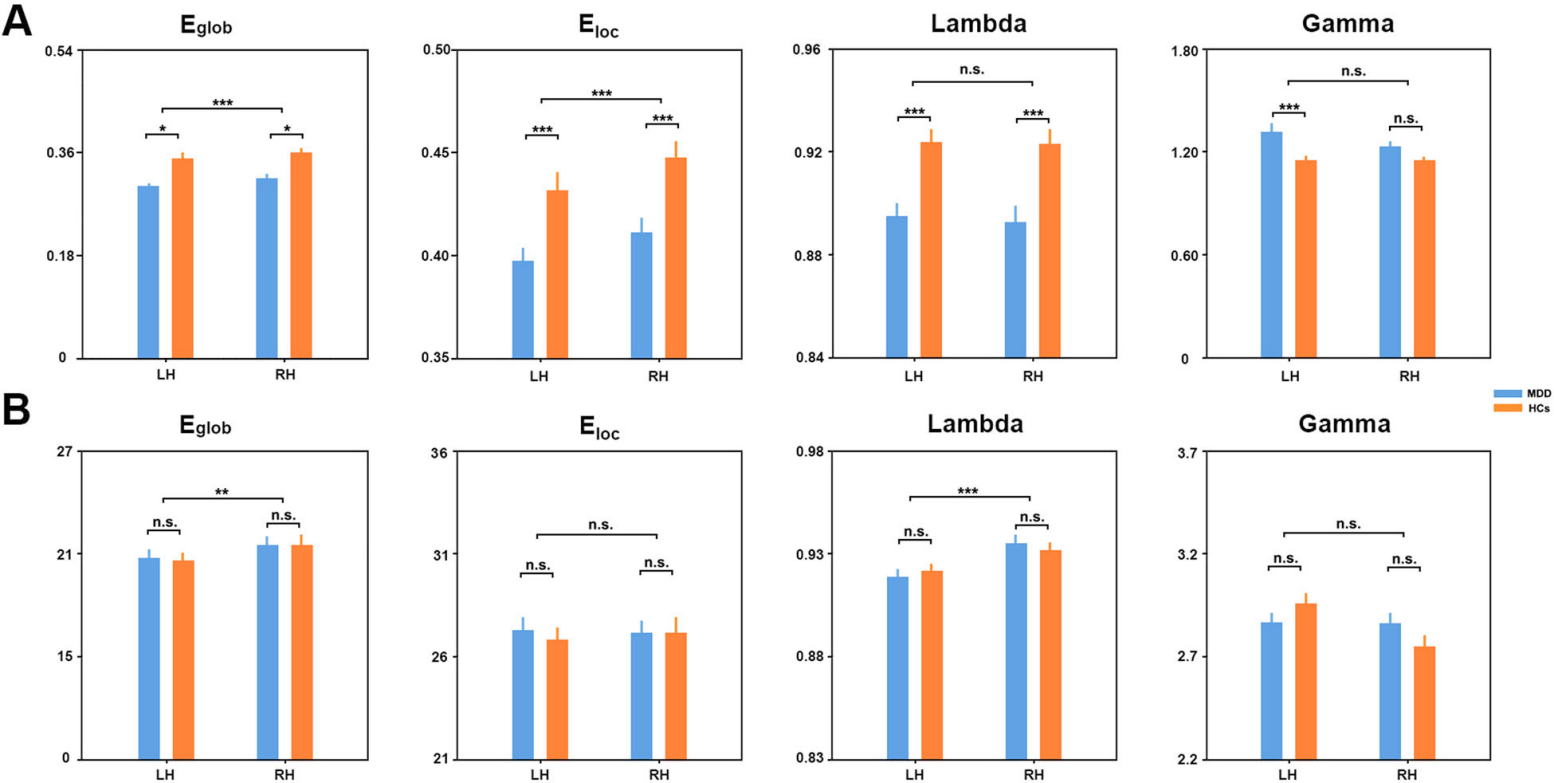
Hemispheric brain network efficiency. Alterations of hemispheric network efficiency for functional (**a**) and structural (**b**) brain networks. MDD major depressive disorder, HCs healthy controls, LH left hemisphere, RH right hemisphere, n.s. non-significant, *P < 0.05; **P < 0.01; ***P < 0.001

Functional networks of both the hemispheres and groups exhibited typical small-world features, that is, higher local efficiency and approximately equal global efficiency to random networks. Nevertheless, statistical analyses revealed that the network efficiency was significantly modulated by hemispheres and differed between groups. Specifically, significant group effects were observed in global efficiency (*F*_1, 68_ = 11.050, *P* = 0.001), local efficiency (*F*_1, 68_ = 8.504, *P* = 0.004), and normalized global efficiency (*F*_1, 68_ = 10.470, *P* = 0.002), and significant hemisphere effects were observed in global efficiency (*F*_1, 68_ = 11.448, *P* = 0.001) and local efficiency (*F*_1, 68_ = 17.059, *P* < 0.001) (Fig. 2a). Post hoc analyses showed that all main effects were due to decreases in the MDD patients vs. HCs for the group factor (all *P* < 0.001) and lower values in the left vs. right hemisphere for the hemisphere factor (all *P* < 0.005). In addition, a significant hemisphere × group interaction was found in normalized local efficiency (*F*_1, 68_ = 4.981, *P* = 0.029) (Fig. 2a). The interaction was attributed to MDD-related increases only in the left hemispheric networks (*T*_63_ = 2.790, *P* = 0.007).

#### Structural networks

Similarly to functional networks, structural networks exhibited typical small-world features regardless of the hemispheres and groups. Further statistical analyses revealed that structural networks were significantly modulated by the hemisphere but not the group. Specifically, significant hemisphere effects were found in global efficiency (*F*_1,68_ = 16.809, *P* < 0.001) and normalized global efficiency (*F*_1,68_ = 11.407, *P* = 0.001), with larger values for the right hemispheric networks (both *P* < 0.005) (Fig. 2b). No significant group effects were found in any global network measures (*P* > 0.05). Again, a significant hemisphere × group interaction (*F*_1, 68_ = 5.832, *P* = 0.018) was found in the normalized local efficiency due to the opposite direction of the between-group differences between the two hemispheric networks (Fig. 2b).

### Regional intra- and inter-hemispheric nodal degree

#### Functional networks

**Fig. 3:**
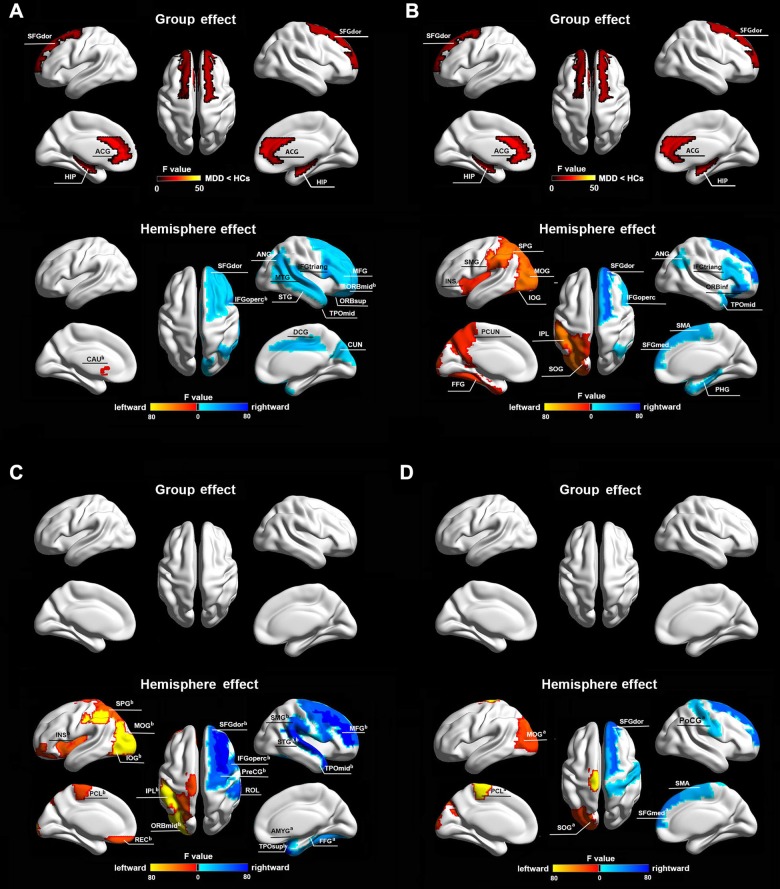
Intra- and inter-hemispheric nodal degree. Alterations of intra-hemispheric (**a**, **c**) and inter-hemispheric (**b**, **d**) nodal degree for functional (**a**, **b**) and structural (**c**, **d**) brain networks. Regions showing significant effects were mapped onto the brain surface with the BrainNet Viewer (Xia et al., 2013). See Supplementary Table 2 for abbreviations of these regions. MDD major depressive disorder, HCs healthy controls. ^a^Regions with significant effects after correcting for regional size. ^b^Regions with significant effects after correcting for different network densities

Significant group effects were observed in the dorsolateral superior frontal gyrus, anterior cingulate gyrus and hippocampus for both intra- and inter-hemispheric nodal degree (*P* < 0.05, Bonferroni-corrected). For hemisphere effects, thirteen and eighteen regions were found for intra- and inter-hemispheric nodal degree, respectively (*P* < 0.05, Bonferroni-corrected) (Fig. 3a, b; Table 1). No regions exhibited significant hemisphere × group interaction effects (*P* > 0.05).

**Table 1:** Regions showing significant effects on intra- and inter-hemispheric nodal degree

	Intra-hemispheric degree			Inter-hemispheric degree		
	Regions	*F*_1,68_	Effect	Regions	*F*_1,68_	Effect
Functional networks	SFGdor	13.339	MDD < HCs	SFGdor	12.106	MDD < HCs
	ACG	17.817	MDD < HCs	ACG	22.000	MDD < HCs
	HIP	13.599	MDD < HCs	HIP	13.606	MDD < HCs
	SFGdor	17.786	RH > LH	SFGdor	53.114	RH > LH
	ORBsup	18.661	RH > LH	IFGoperc	20.895	RH > LH
	MFG	12.548	RH > LH	IFGtriang	17.826	RH > LH
	ORBmid^b^	21.211	RH > LH	ORBinf	40.904	RH > LH
	IFGoprec^b^	31.136	RH > LH	SMA	22.392	RH > LH
	IFGtriang	16.421	RH > LH	SFGmed	26.374	RH > LH
	DCG	21.929	RH > LH	PHG	22.392	RH > LH
	CUN	17.388	RH > LH	ANG	14.002	RH > LH
	ANG	21.693	RH > LH	TPOmid	24.330	RH > LH
	STG	23.075	RH > LH	INS	13.977	LH > RH
	MTG	15.817	RH > LH	SOG	27.896	LH > RH
	TPOmid	17.138	RH > LH	MOG	78.634	LH > RH
	CAU^b^	11.690	LH > RH	IOG	36.300	LH > RH
				FFG	16.921	LH > RH
				SPG	31.935	LH > RH
				IPL	70.614	LH > RH
				SMG	27.027	LH > RH
				PCUN	13.147	LH > RH
Structural networks	PreCG^b^	48.361	RH > LH	SFGdor	39.537	RH > LH
	SFGdor^b^	52.801	RH > LH	SMA	22.652	RH > LH
	MFG^b^	113.355	RH > LH	SFGmed^a^	40.286	RH > LH
	IFGoperc^b^	52.946	RH > LH	PoCG^a^	20.461	RH > LH
	ROL	17.230	RH > LH	SOG^a^	19.968	LH > RH
	AMYG^a^	17.766	RH > LH	MOG^a^	21.061	LH > RH
	FFG^a^	32.460	RH > LH	PCL^a^	99.983	LH > RH
	SMG^b^	56.142	RH > LH			
	STG^a,b^	74.004	RH > LH			
	TPOsup^b^	51.968	RH > LH			
	TPOmid^b^	72.701	RH > LH			
	ORBmid^b^	16.715	LH > RH			
	REC^b^	15.444	LH > RH			
	INS^b^	20.886	LH > RH			
	MOG^b^	168.293	LH > RH			
	IOG^b^	78.039	LH > RH			
	SPG^b^	19.039	LH > RH			
	IPL^b^	98.392	LH > RH			
	PCL^b^	13.315	LH > RH			

*MDD* major depressive disorder, *HCs* healthy controls, *LH* left hemisphere, *RH* right hemisphere

^a^Regions with significant effects after correcting for regional size

^b^Regions with significant effects after correcting for different network densities. See Supplementary Table 2 for abbreviations of these regions

#### Structural networks

Significant hemisphere effects were observed in nineteen regions for the intra-hemispheric nodal degree and seven regions for the inter-hemispheric nodal degree (*P* < 0.05, Bonferroni-corrected) (Fig. 3c, d; Table 1). No regions showed significant group or hemisphere × group interaction effects (*P* > 0.05).

### Functional–structural Coupling

**Fig. 4:**
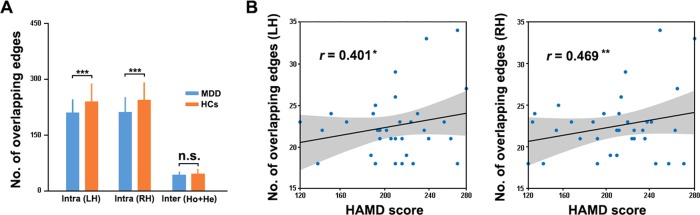
Functional-structural connectivity overlapping. Decreased numbers of functional–structural overlapping for intra- hemispheric connections in MDD (**a**) and their relationships with disease severity of patients (**b**). LH left hemisphere, RH right hemisphere, HAMD Hamilton Depression Scale

A significant group (*F*_1,68_ = 9.303, *P* = 0.003) but not hemisphere (*F*_1,68_ = 0.734, *P* = 0.395) or group × hemisphere interaction (*F*_1,68_ = 0.179, *P* = 0.673) was observed for the amount of functional–structural overlapping for the intra-hemispheric connections, which was due to a decrease in the patients compared with the HCs (T_135_ = 4.444, *P* < 0.001) (Fig. 4a). Regarding the amount of functional–structural overlapping for inter-hemispheric connections, no significant between-group differences were found (*T*_65_ = 1.073, *P* = 0.287). Regarding the functional–structural coupling degree, positive correlations were found for both the intra-hemispheric (left: MDD = 0.318 ± 0.068 and HCs = 0.312 ± 0.061; right: MDD = 0.322 ± 0.067 and HCs = 0.328 ± 0.054) and inter-hemispheric (MDD = 0.250 ± 0.138 and HCs = 0.228 ± 0.163) connections with no significant between-group differences (all *P* > 0.05).

### Brain-clinical Correlation

Within the MDD group, the functional–structural coupling in the number of overlapping intra-hemispheric connections exhibited significantly positive correlations with the HAMD scores of the patients (left: *r* = 0.401, *P* = 0.023; right: *r* = 0.469, *P* = 0.007) (Fig. 4b). No significant correlations were found between other network measures and clinical variables.

### Validation and reproducibility

#### Effects of regional size

No significant effects of group, hemisphere or group × hemisphere interaction were observed in the correlations between regional size and nodal degree (intra-hemispheric or inter-hemispheric) for the functional networks (*P* > 0.05). However, significant hemisphere effects were observed on the correlations between regional size and intra-hemispheric (*F*_1,68_ = 52.375, *P* < 0.001) and inter-hemispheric (*F*_1,68_ = 52.375, *P* < 0.001) nodal degree for the structural networks. After correcting for regional sizes, three out of nineteen for intra-hemispheric nodal degree and five out of seven for inter-hemispheric nodal degree remained to show significant hemisphere effects (*P* < 0.05, Bonferroni-corrected) (Fig. 3c, d, bottom; Table 1).

#### Effects of thresholding procedure

Using the sparsity-based thresholding procedure, no significant between-group differences were found in any network measure for either functional or structural networks (*P* > 0.05, corrected as needed). Regarding the hemisphere factor, significant effects were reproduced for global efficiency of the functional networks (*F*_1,68_ = 10.080, *P* = 0.002) and normalized global efficiency of the structural networks (*F*_1,68_ = 7.843, *P* = 0.007). Regionally, three out of thirteen for the functional networks and sixteen out of nineteen for the structural networks remained to show significant hemisphere effects on intra-hemispheric nodal degree (*P* < 0.05, Bonferroni-corrected) (Fig. 3a, c, bottom; Table 1).

## Discussion

In this study, we combined R-fMRI and DTI with graph-based network approaches to investigate functional and structural hemispheric networks in patients with MDD. The main findings are summarized as follows: (1) At the connectional level, the MDD patients exhibited significantly decreased intra-hemispheric (both left and right) and inter-hemispheric (heterotopic) connectivity for functional but not structural networks; both functional and structural networks exhibited rightward advantages for intra-hemispheric connectivity. (2) At the global level, despite common small-world organization for both the hemispheres and imaging modalities, only functional networks exhibited topological disruptions in the patients; both functional and structural networks were wired more efficiently for the right than left hemisphere. (3) At the nodal level, only functional networks presented MDD-related decreases in both intra- and inter-hemispheric nodal degree in the SFGdor, ACG, and HIP; multiple frontal and temporal regions showed rightward advantages and occipital and parietal sites showed leftward advantages irrespective of imaging modalities and connectional types. (4) Functional–structural couplings were significantly decreased for intra-hemispheric connections in MDD, and these decreases were related to the disease severity of the patients.

### Decreased intra- and inter-hemispheric functional connectivity in MDD

We found that the functional networks of the MDD patients showed decreased intra- and inter-hemispheric connectivity compared with the HCs. This finding suggests impaired intra-hemispheric integration and inter-hemispheric communication in MDD. MDD-related disruptions of functional connectivity have been frequently reported for multiple distributed brain systems, such as the default mode network and frontoparietal network^33^. With the hemisphere taken into consideration, our findings suggest that the disruptions are involved in not only intra-hemispheric but also inter-hemispheric functional connectivity. Notably, inconsistent with previous studies that have consistently reported MDD-related decreases in homotopic functional connectivity^34–39^, we only observed a trend toward significant decreases in MDD. This discrepancy may be due to the different spatial scales at which the network analyses were conducted (i.e., region level vs. voxel level). Overall, our findings together with those of previous studies collectively propose that functional disconnection appears to be a reliable marker for MDD. However, we did not find MDD-related alterations in structural connectivity regardless of connection type. In line with our results, a previous study showed that MDD was associated with reduced inter-hemispheric homotopic functional connectivity but had intact callosal fiber pathways and normal asymmetries in gray matter volumes^36^. These findings imply that additional mechanisms are implicated in the decreased functional connectivity in MDD. It is worth noting that several previous studies have reported altered structural connectivity in MDD^2,40–42^. Factors such as highly heterogeneous patients and methodological variants may contribute to the inconsistency^6^.

Regarding asymmetry, we found that functional and structural networks exhibited significant rightward advantages for intra-hemispheric connectivity. This finding is consistent with a previous structural network study showing that the right hemisphere is more densely interconnected than the left hemisphere^21^. The right-greater-than-left intra-hemispheric connectivity supports the previous assumption that the right hemisphere is more related to general processes, while the left hemisphere is relatively specific^43^.

### Altered efficiency of functional hemispheric networks in MDD

Our network efficiency analysis revealed that both functional and structural hemispheric networks exhibited small-world organization in both groups. This finding is consistent with previous studies indicating that each hemispheric network has evolved into a highly optimized system in favor of efficient information processing^21,23,44^. However, only functional networks (both hemispheres) showed reduced global and local efficiency in the patients. This finding is in agreement with previous whole-brain network analyses of MDD^4,45^. The decreases imply disrupted segregation and integration to support modular and distributed parallel information processing for functional hemispheric networks in MDD. Moreover, the global efficiency decrease held for both hemispheres after normalization by random networks, suggesting intrinsic impairments of global integration in patients. Interestingly, only the left hemisphere showed increased normalized local efficiency in the MDD patients, indicating hemisphere-dependent alterations in the degree of optimization for local functional segregation. Given that the small-world parameters reflect an optimal balance between local specialization and global integration, our results indicate a disturbance of the normal balance for functional networks in MDD that is dependent on the hemisphere. These findings contribute novel insights into network dysfunction in MDD.

No significant alterations were observed in the network efficiency for structural hemispheric networks in the patients. This finding is in line with our structural connectivity results and previous structural network studies demonstrating preserved global network configurations in MDD^41,46–48^. Notably, altered global organization of structural brain networks was also reported in MDD^2,49^. Further studies are needed to uncover potential factors contributing to the inconsistent findings.

With regard to hemispheric lateralization, rightward asymmetries in network efficiency were observed for functional and structural networks in both groups, suggesting a more efficient wiring layout in the right hemisphere. The rightward asymmetry of the network efficiency is not surprising given that the right hemisphere is more densely interconnected, as revealed by our intra-hemispheric connectivity analysis and that reported in a previous study^21^. Interestingly, the rightward advantage of brain networks emerges during adolescence^24^, exists in other species^21^ and is related to psychological disorders^25^. Thus, network asymmetry may serve as a promising avenue for examining cross-species brain evolution and for revealing shared network mechanisms among brain disorders.

### Decreased regional centrality of functional hemispheric networks

In this study, we used intra- and inter-hemispheric degree to capture the regional roles in coordinating within-hemispheric integration and between-hemispheric communication. Using these two measures, the bilateral SFG, ACG, and HIP were consistently observed to show MDD-related decreases in functional networks. Impaired functional connectivity of these regions has been frequently reported in previous MDD studies^3,50–52^. Here, our results further indicate that the impaired functional connectivity is involved in regions not only in their ipsilateral but also contralateral hemispheres, which are therefore proposed as key functional disconnection nodes in MDD. Considering the crucial role of the SFG, ACG, and HIP in emotion regulation and self-awareness^51,53^, the observed functional connectivity impairments may be implicated in the pathogenesis of MDD that causes dysfunction of cognitive and emotional processing in the disease. Again, no regions showed MDD-related alterations in nodal degree for structural hemispheric networks. This finding is consistent with a previous study showing that the anatomical connectivity profile of the brain partly shapes its functional repertoire in a location-dependent manner^54^.

From the perspective of asymmetry, intra-, and inter-hemispheric nodal degree revealed similar patterns between functional and structural networks for both intra- and inter-hemisphere nodal degree. These results, together with the findings derived from the overall connectivity and network efficiency analyses, collectively suggest that network asymmetry is a shared and comparable organizational principle between functional and structural networks in the human brain. Although a detailed discussion of the observed regional asymmetries is beyond the scope of the current study, presumably, their biological role is to support hemispheric lateralization of specific cognitive function of the human brain. For example, consistent with a previous study^22^, we found that the INS exhibited a leftward advantage in its regional connectivity profiles. Given the central role of the INS in word processing^55,56^, its left-more-than-right connectivity may account, at least in part, for the left lateralization of word processing of the human brain. It should be highlighted that existing findings are controversial with respect to the regional asymmetry of structural and functional connectivity. For instance, in contrast to our results, a rightward advantage was reported for the INS in another previous study^23^. The inconsistency might be due to differences in image processing pipelines and/or biological factors, such as age, sex, and handedness, across studies. Recently, Kong and colleagues provided the most credible results regarding local cortical asymmetry by analyzing 17,141 healthy individuals worldwide^57^. Similar large-sample studies are warranted in the future to provide a reliable reference to assist in clarifying controversial findings for hemispheric asymmetry of regional connectivity profiles.

### Decreased functional–structural hemispheric coupling and its clinical relevance in MDD

Functional–structural coupling is an important topic in network neuroscience^18^. Focusing on the coupling degree, several previous studies consistently show positive correlations between functional and structural connectivity at both single-circuit^14,15^ and whole-brain^8,16,29^ levels. Nevertheless, recent evidence also highlights that the functional–structural coupling degree is dependent on the state of functional networks^58^ and that there is a poor correspondence between functional and structural connectivity patterns^59^. Accordingly, distinct network organizations are increasingly reported between functional and structural brain networks^60,61^, which support our findings of functional and structural separation in revealing MDD-related network alterations. Here, we found that the amount rather than the degree was decreased in the patients. This finding implies a worse correspondence between functional and structural connectivity patterns but a preserved coupling degree for the overlapping connections in MDD. Given our results that functional rather structural networks were disrupted in MDD, the decreased coupling amount presumably is a consequence of impaired functional interactions but intact structural pathways between regions. Interestingly, the decreased functional–structural coupling amount of intra-hemispheric connectivity in MDD exhibited significantly positive correlations with the disease severity (i.e., HAMD score) of the patients. This finding implies that for the MDD patients, higher HAMD scores are associated with stronger functional–structural couplings for their hemispheric networks. The counterintuitive brain network-behavioral relationships have been previously reported in MDD^3^ and other diseases, such as attention-deficit/hyperactivity disorder^62^ and schizophrenia^63^, indicating a common phenomenon in brain disorders. Although the counterintuitive relationships are poorly understood, a commonly accepted interpretation is that they may reflect compensatory reorganization of the patients’ brains in response to atypical conditions. Despite the lack of direct evidence, this speculation is plausible given the highly plastic nature of the human brain. Notably, positive correlations were observed for the two hemispheres, implying a common mechanism by which MDD exerts an influence on the functional–structural couplings of brain networks.

### Limitations

This study has several limitations that may affect the reproducibility of our results. First, the sample size is relatively small. Furthermore, the patients were clinically heterogeneous with respect to the number of episodes, disease duration and age of onset. These factors may weaken the sensitivity in revealing MDD-related alterations in hemispheric networks. Second, there are several methodological issues that may also affect the reproducibility of the current findings, such as node definition^30,64^, connectivity estimation^65,66^, and thresholding procedure^31,67^. It is important in the future to identify robust network alterations in MDD that are independent of various choices of these factors. Third and finally, a deterministic tractography method was utilized to construct structural brain networks, which suffers from problems in crossing and long-distance fiber tracking. Such problems may result in false-positive discoveries of interregional structural connectivity and further confound the topological quantification of structural networks and functional–structural coupling. Future studies are required to test the potential effects by employing different and, in particular, newly developed tractography methods.

## Conclusion

In conclusion, the present study investigated large-scale brain network alterations in MDD by taking network modality and brain hemisphere into account. We found that functional brain networks exhibited significant degeneration and reorganization at multiple levels, while structural brain networks were largely intact in patients with MDD. These findings lend support to views of MDD as a network dysfunctional syndrome and indicate that network dysfunction is essentially functional rather than a derivative of structural abnormalities. Moreover, some of the functional alterations were shared, while others were dependent on the hemisphere, suggesting common and specific mechanisms of the two hemispheres affected by MDD. In particular, the dorsolateral superior frontal gyrus, anterior cingulate gyrus, and hippocampus exhibited decreased functional connectivity in both intra-hemispheric integration and inter-hemispheric communication in the patients. Thus, these regions may be key functional disconnection nodes in MDD and serve as promising candidates for therapeutic targets that can be addressed to improve the prognosis of the disease. Last but not least, we found decreased functional–structural coupling in MDD, which was related to the disease severity of the patients. This finding implies the potential of functional–structural network coupling as a promising biomarker for monitoring disease progression of MDD. Overall, this study demonstrates modality- and hemisphere-dependent and invariant network alterations in MDD, which are helpful for establishing elaborate and characteristic patterns of integrative dysfunction in this disease.

## Supplementary information

supplemental material
