# Supplementary material for: Connectome analysis of functional and structural hemispheric brain networks in major depressive disorder
Source: Transl Psychiatry. 2019 Apr 12;9:136. doi: 10.1038/s41398-019-0467-9 (PMC6461612; doi:10.1038/s41398-019-0467-9)
Supplement: Supplementary file 1 — supplemental material [file 41398_2019_467_MOESM1_ESM.docx]

**Supplementary Material**

**MRI Data Acquisition**

*R-fMRI data*. R-fMRI data were obtained using a single-shot, gradient-recalled echo-planar imaging sequence parallel to the line of the anterior-posterior commissure. The acquisition parameters were as follows: 37 axial slices; repetition time (TR) = 2000 ms; echo time (TE) = 30 ms; thickness/gap = 3.2/0 mm; flip angle (FA) = 90°; field of view (FOV) = 220×220 mm^2^; and matrix = 128×128. A total of 184 volumes were acquired for each participant.

*DTI data*. DTI data were acquired using a single-shot echo-planar imaging-based sequence: TR = 8300 ms; TE = 84 ms; thickness/gap = 3.2/0 mm; FA = 90°; FOV = 220×220 mm^2^; matrix = 128×128; 48 diffusion gradient directions; and 8 b0 images (1000 s/mm^2^).

*Structural MRI data*. High-resolution T1-weighted images were also acquired for each participant to facilitate image registration with a three-dimensional spoiled gradient-recalled sequence: 176 axial slices; TR = 8.1 ms; TE = 3.1 ms; thickness/gap = 1.0/0 mm; FA = 8°; FOV = 250×250 mm^2^; and matrix = 256×256.

**Data Preprocessing**

*R-fMRI data*. After discarding the first five volumes, individual functional images were corrected for intra-volume temporal offsets (Sinc interpolation) and inter-volume head motion (rigid-body transformation). No participants were excluded in terms of the criterion of a displacement > 2.5 mm or an angular rotation > 2.5° in any direction. Moreover, several summary scalars of the head motion parameters, including the maximum, root mean square and mean framewise displacement, did not significantly differ between the two groups (*P* > 0.05). The corrected images were then spatially normalized to the standard Montreal Neurological Institute (MNI) space using transformation fields derived from tissue segmentation of individual T1 images and resampled to 3-mm isotropic voxels. The normalized images further underwent removal of linear trend and temporal band-pass filtering (0.01 - 0.1 Hz). Finally, several nuisance signals, including 24-parameter head motion profiles^1^, white matter signals and cerebrospinal fluid signals, were regressed out from each voxel’s time series.

*DTI data.* The diffusion-weighted images were first corrected for distortions caused by eddy currents and for head motions through an affine registration to the b0 images. The diffusion tensor models were then estimated at each voxel, and diagonalization was performed to yield three eigenvalues and eigenvectors^2^. Whole-brain white matter fiber tracts were subsequently reconstructed in native diffusion space for each participant using a continuous streamline-tracking algorithm^3^. The tractography was terminated when it reached a voxel with an FA less than 0.2 or when the turning angle was greater than 45˚ between adjacent voxels.

**Construction of Functional and Structural Networks**

*Node definition*. To define brain network nodes, we employed an automated anatomical labeling (AAL) atlas^4^ to parcel the cerebrum into 90 regions of interest (ROIs, 45 in each hemisphere) (Table 2). This atlas was used to construct both functional and structural brain networks to enable examinations of functional-structural coupling. Notably, the cerebellum was excluded from this study to maximize the comparability of our results with previous findings with respect to node definition^5^.

*Edge definition*. To define brain network edges, we calculated pairwise interregional Pearson correlation coefficients for the R-fMRI data and pairwise fiber numbers for the DTI data. Specifically, for the R-fMRI data, the mean time series was first extracted for each ROI by averaging the signals across all voxels within it. The resultant mean time series were subsequently correlated with each other to generate a 90 × 90 correlation matrix for each participant. Finally, a significance threshold (*P* < 0.05, Bonferroni corrected) was used to exclude non-significant elements in the matrices. The significance-based thresholding procedure can effectively avoid erroneous evaluations of network topology^6^ and has been used in previous brain network studies^7,8^. Notably, negative correlations were also excluded in this study because of their ambiguous interpretation^9-11^ and detrimental effects on test-retest reliability^12^. Regarding the DTI data, for each participant, an affine transformation (co-registration of each participant’s FA image in native diffusion space to the corresponding T1 image) and a non-linear transformation (registration of each participant’s T1 image to the ICBM152 T1 template in the MNI space) were first estimated. Based on these two transformations, an inverse transformation was then calculated and used to warp the AAL atlas from the MNI space to each participant’s native diffusion space. Combined with the whole-brain white matter fiber tracts derived above, we calculated the number of fiber streamlines with their two endpoints located in any pair of regions. This procedure resulted in a 90 × 90 fiber number weighted structural connectivity matrix for each participant. To exclude potential noise, two regions were considered structurally connected only if at least three fiber streamlines existed between them^13^.

After these procedures, we obtained two whole-brain weighted networks for each participant. These networks were further divided into two 45 × 45 hemispheric networks by eliminating all inter-hemispheric connections.

**Graph-based Network Analysis**

*Global network efficiency*. In this study, network efficiency metric was used to characterize parallel information processing of the functional and structural hemispheric networks derived above at the global and local levels^14,15^. Mathematically, for a network *G* with *N* nodes, global efficiency is defined as follows:

$E_{\mathrm{glob}}\left( G \right)=\frac{1}{N\left( N-1 \right)}\sum_{i\neq j\in G} \frac{1}{d_{\mathrm{ij}}}$,

where is the shortest path length between node *i* and node *j* and is calculated as the smallest sum of edge length (i.e., the reciprocal of the edge weight: correlation coefficient or fiber number) over all possible paths from node *i* to node *j*. Global efficiency reflects how efficiently information can be exchanged over a network, considering a parallel system in which each node sends information concurrently along the network. Local efficiency is calculated as follows:

$E_{\mathrm{loc}}\left( G \right)=\frac{1}{N}\sum_{i\in G} E_{\mathrm{glob}}\left( G_{i} \right)$,

where $E_{\mathrm{glob}}\left( G_{i} \right)$ is the global efficiency of $G_{i}$, the subgraph composed of the neighbors of node *i* (i.e., nodes linked directly to node *i*). The local efficiency reflects the extent to which a network is fault-tolerant by measuring the mean capability of information exchange over its subgraphs when the index nodes are eliminated.

To estimate the small-world organization, global efficiency and local efficiency derived from real brain networks were normalized by dividing them by the corresponding mean of 100 matched random networks. The random networks were generated using a random rewiring procedure that preserves the same number of nodes and edges and the same degree distribution as real brain networks^16^. Typically, a small-world network has a normalized global efficiency approximately equal to 1 and a normalized local efficiency greater than 1^17,18^.

*Regional nodal centrality.* To characterize nodal attributes of each region, we calculated weighted nodal degree, which is defined as the sum of weights (i.e., correlation coefficient or fiber number) over connections linked to a node. Specifically, for a given node *i*, the summation was restricted to connections linking the node *i* and nodes of its ipsilateral hemisphere to derive the intra-hemispheric degree and connections linking the node *i* and nodes of its contralateral hemisphere to derive the inter-hemispheric degree.

**References**

1. Friston KJ, Williams S, Howard R, Frackowiak RS, Turner R. Movement-related effects in fMRI time-series. *Magnetic resonance in medicine* 1996; 35(3): 346-355.

2. Basser PJ, Pierpaoli C. Microstructural and physiological features of tissues elucidated by quantitative-diffusion-tensor MRI. *Journal of magnetic resonance* Series B 1996; 111(3): 209-219.

3. Mori, S. *et al*. Three‐dimensional tracking of axonal projections in the brain by magnetic resonance imaging. *Annals of Neurology* 1999; 45: 265-269.

4. Tzourio-Mazoyer N, Landeau B, Papathanassiou D, Crivello F, Etard O, Delcroix N, *et al*. Automated anatomical labeling of activations in SPM using a macroscopic anatomical parcellation of the MNI MRI single-subject brain. *NeuroImage* 2002; 15(1): 273-289.

5. Gong Q, He Y. Depression, neuroimaging and connectomics: a selective overview. *Biological psychiatry* 2015; 77(3): 223-235.

6. Toppi J, De Vico Fallani F, Vecchiato G, Maglione AG, Cincotti F, Mattia D, *et al*. How the statistical validation of functional connectivity patterns can prevent erroneous definition of small-world properties of a brain connectivity network. *Computational and mathematical methods in medicine* 2012; 2012: 130985.

7. Wang J, Zuo X, Dai Z, Xia M, Zhao Z, Zhao X, *et al*. Disrupted functional brain connectome in individuals at risk for Alzheimer's disease. *Biological psychiatry* 2013; 73(5): 472-481.

8. Zuo XN, Ehmke R, Mennes M, Imperati D, Castellanos FX, Sporns O, *et al*. Network centrality in the human functional connectome. *Cerebral cortex* 2012; 22(8): 1862-1875.

9. Fox MD, Corbetta M, Snyder AZ, Vincent JL, Raichle ME. Spontaneous neuronal activity distinguishes human dorsal and ventral attention systems. *Proceedings of the National Academy of Sciences of the United States of America* 2006; 103(26): 10046-10051.

10. Murphy K, Birn RM, Handwerker DA, Jones TB, Bandettini PA. The impact of global signal regression on resting state correlations: are anti-correlated networks introduced? *NeuroImage* 2009; 44(3): 893-905.

11. Weissenbacher A, Kasess C, Gerstl F, Lanzenberger R, Moser E, Windischberger C. Correlations and anticorrelations in resting-state functional connectivity MRI: a quantitative comparison of preprocessing strategies. *NeuroImage* 2009; 47(4): 1408-1416.

12. Wang JH, Zuo XN, Gohel S, Milham MP, Biswal BB, He Y. Graph theoretical analysis of functional brain networks: test-retest evaluation on short- and long-term resting-state functional MRI data. *PloS one* 2011; 6(7): e21976.

13. Shu N, Liu Y, Li K, Duan Y, Wang J, Yu C, *et al*. Diffusion tensor tractography reveals disrupted topological efficiency in white matter structural networks in multiple sclerosis. *Cerebral cortex* 2011; 21(11): 2565-2577.

14. Latora V, Marchiori M. Efficient behavior of small-world networks. *Physical review letters* 2001; 87(19): 198701.

15. Latora V, Marchiori M. Economic small-world behavior in weighted networks. *The European Physical Journal B - Condensed Matter and Complex Systems* 2003; 32(2): 249-263.

16. Maslov S, Sneppen K. Specificity and stability in topology of protein networks. *Science* 2002; 296(5569): 910-913.

17. Wang J, Wang L, Zang Y, Yang H, Tang H, Gong Q, *et al*. Parcellation-dependent small-world brain functional networks: a resting-state fMRI study. *Human brain mapping* 2009; 30(5): 1511-1523.

18. Watts DJ, Strogatz SH. Collective dynamics of 'small-world' networks. *Nature* 1998; 393(6684): 440-442.

**Supplementary Table 1.** Demographics, clinical characteristics and head motion of all participants

|  | MDD (n = 35) | HCs (n = 35) | *P*-value |
| --- | --- | --- | --- |
| Gender (F/M) | 22/13 | 23/12 | 0.803 |
| Age (years) | 44.400 ± 14.034 | 41.029 ± 12.871 | 0.298 |
| Education level (years) | 9.957 ± 5.339 | 11.886 ± 4.880 | 0.119 |
| Handedness (R/L) | 35/0 | 35/0 | - |
| HAMD | 22.514 ± 3.807 | - | - |
| Disease duration (years) | 9.871 ± 5.330 | - | - |
| Age of onset (years) | 34.500 ± 17.449 | - | - |
| Number of episodes | 1.543 ± 0.981 | - | - |
| Duration of current episode (years) | 1.602 ± 1.913 | - | - |
| Maximum of head motion | 0.103 ± 0.062 | 0.114 ± 0.057 | 0.460 |
| Root mean square of head motion | 0.766 ± 0.524 | 0.923 ± 0.532 | 0.219 |
| Mean framewise displacement of head motion | 0.202 ± 0.121 | 0.210 ± 0.095 | 0.778 |

Data are presented as mean ± standard deviation. MDD, major depressive disorder; HCs, healthy controls; HAMD, Hamilton Rating Scale for Depression; M, male; F, female; R, right; L, left.

**Supplementary Table 2.** Regions of interest

| **Index** | **Regions** | **Abbreviations** | **Index** | **Regions** | **Abbreviations** |
| --- | --- | --- | --- | --- | --- |
| 1,2 | Precentral gyrus | PreCG | 47,48 | Lingual gyrus | LING |
| 3,4 | Superior frontal gyrus, dorsolateral | SFGdor | 49,50 | Superior occipital gyrus | SOG |
| 5,6 | Superior frontal gyrus, orbital part | ORBsup | 51,52 | Middle occipital gyrus | MOG |
| 7,8 | Middle frontal gyrus | MFG | 53,54 | Inferior occipital gyrus | IOG |
| 9, 10 | Middle frontal gyrus, orbital part | ORBmid | 55,56 | Fusiform gyrus | FFG |
| 11,12 | Inferior frontal gyrus, triangular part | IFGoperc | 57,58 | Postcentral gyrus | PoCG |
| 13,14 | Inferior frontal gyrus, triangular part | IFGtriang | 59,60 | Superior parietal gyrus | SPG |
| 15,16 | Inferior frontal gyrus, orbital part | ORBinf.L | 61,62 | Inferior parietal, but supramarginal and angular gyri | IPL |
| 17,18 | Rolandic operculum | ROL | 63,64 | Supramarginal gyrus | SMG |
| 19,20 | Supplementary motor area | SMA | 65,66 | Angular gyrus | ANG |
| 21,22 | Olfactory cortex | OLF | 67,68 | Precuneus | PCUN |
| 23,24 | Superior frontal gyrus, medial | SFGmed | 69,70 | Paracentral lobule | PCL |
| 25,26 | Superior frontal gyrus, medial orbital | ORBsupmed | 71,72 | Caudate nucleus | CAU |
| 27,28 | Gyrus rectus | REC | 73,74 | Lenticular nucleus, putamen | PUT |
| 29,30 | Insula | INS | 75,76 | Lenticular nucleus, pallidum | PAT |
| 31,32 | Anterior cingulate and paracingulate gyri | ACG | 77,78 | Thalamus | THA |
| 33,34 | Median cingulate and paracingulate gyri | DCG | 79,80 | Heschl gyrus | HES |
| 35,36 | Posterior cingulate gyrus | PCG | 81,82 | Superior temporal gyrus | STG |
| 37,38 | Hippocampus | HIP | 83,84 | Temporal pole: superior temporal gyrus | TPOsup |
| 39,40 | Parahippocampal gyrus | PHG | 85,86 | Middle temporal gyrus | MTG |
| 41,42 | Amygdala | AMYG | 87,88 | Temporal pole: middle temporal gyrus | TPOmid |
| 43,44 | Calcarine fissure and surrounding cortex | CAL | 89,90 | Inferior temporal gyrus | ITG |
| 45,46 | Cuneus | CUN |  |  |  |

Regions of the left and right hemispheres are indexed by odd and even numbers, respectively.
